# Skeletal changes of the axial axis and lower limbs in overweight children and adolescents

**DOI:** 10.1590/1984-0462/2023/41/2021298

**Published:** 2023-04-07

**Authors:** Pietra Luz Moleirinho Lima, Mateus de Paiva Breziniscki, Guilherme Henrique Pedrassoli, Edilson Forlim, Jamil Faissal Soni, Rosana Bento Radominski, Suzana Nesi França, Julienne Angela Ramires de Carvalho

**Affiliations:** a Universidade Federal do Paraná, Curitiba, PR, Brazil.; b Pontifícia Universidade Católica do Paraná, Curitiba, PR, Brazil.

**Keywords:** Childhood obesity, Postural evaluation, Skeletal changes, Overweight, Obesidade infantil, Avaliação postural, Alterações esqueléticas, Sobrepeso

## Abstract

**Objective:**

To evaluate the presence of axial skeletal deviations in children and adolescents and to relate them to body mass index (BMI), age and sex.

**Methods:**

101 patients aged 7 to 17 years old were included in this study; exclusion criteria were primary orthopedic diseases and syndromes or treatments that affect growth. Patients were grouped according to their BMI Z-score: eutrophic (n=29), overweight (n=18) and obese (n=54). They underwent static clinical inspection was made by simetrographic technique. Intermaleolar distance was obtained, Adam’s forward bend and tiptoe tests were performed.

**Results:**

When comparing obese and eutrophic patients, changes in the cervical spine (p<0.01), spine (p<0.001), hip (p<0.01) and shoulders (p<0.001) were present in more than half of the obese patients (62.5%, 62.2%, 79.9% and 55.4%, respectively). Changes in the knees were more frequent among obese (p<0.001) when compared to eutrophic patients. There was no variation regarding age or sex (p>0.05).

**Conclusions:**

being overweight influences skeletal deviations in children and adolescents.

## RESUMO

**Objetivo:**

Avaliar a presença de desvios do esqueleto axial em crianças e adolescentes e relacioná-los com índice de massa corpórea, idade e sexo.

**Métodos:**

Fizeram parte deste estudo 101 pacientes de sete a 17 anos, os quais não possuíam doenças ortopédicas primárias, síndromes ou tratamentos que afetassem o crescimento. Os pacientes foram agrupados conforme os escores Z do índice de massa corpórea em: eutróficos (n=29), com sobrepeso (n=18) e obesos (n=54). Foram avaliados por meio da inspeção clínica estática, com simetrógrafo de parede, medida da distância intermaleolar, manobra de Adams e teste da ponta dos pés.

**Resultados:**

Quando comparados os pacientes obesos com os eutróficos, alterações de coluna cervical (p<0,01), coluna (p<0,001), quadril (p<0,01) e ombros (p<0,001) ocorreram em mais da metade dos obesos (62,5, 62,2, 79,9 e 55,4%, respectivamente). Alterações nos joelhos foram mais frequentes entre os obesos (p<0,001) quando comparados aos eutróficos. Não houve variação com a idade ou o sexo (p>0,05).

**Conclusões:**

Conclui-se que o excesso de peso exerce influência sobre desvios do esqueleto em crianças e adolescentes.

## INTRODUCTION

In Brazil, data from the Food and Nutrition Surveillance System of the Ministry of Health showed that, in 2015, 9.7% of all children aged between seven and ten years old were overweight for their age.^1^ When it comes to comorbidities directly related to childhood obesity, the World Health Organization (WHO) emphasizes the increasing number of cases of type 2 diabetes and dyslipidemia, in addition to stating that overweight children are at greater risk of becoming overweight adults.^1,2^ Such complications are well known and studied, but the same is not true for orthopedic complications. These are not routinely assessed during health care, and few studies that relate childhood obesity with orthopedic alterations are available in the national medical literature.^2^

Osteoarticular changes can be present in eutrophic and obese individuals. However, the prevalence is higher in obese adults due to the overload resulting from increased body mass, which causes structural and functional changes.^3^ In the period between 7 and 14 years of age, children are more susceptible to skeletal system changes, because the proportion of collagen in bones higher, and there is greater tolerance to deformation and lower resistance to compression. Such characteristics favor bone deformity, but also facilitate postural correction, which justifies the importance of early diagnosis of postural deviations.^4^

Postural adequacy is important when it comes to individuals in development phase. When physiological spatial relations are altered, muscle imbalances, capsulo-ligamentous structures and misalignment of body segments arise, with consequent adaptive changes of the bones in order to accommodate themselves to the new mechanical conditions imposed. This can induce the appearance of definitive deformities in adult life, impair the ability to perform physical activity, directly affect the quality of life of these patients and contribute to the maintenance or worsening of obesity.^5^

The main objective of the study was to evaluate deviations of the axial skeleton in children and adolescents assisted at the Pediatric Endocrinology Unit (UEP) of the Hospital de Clínicas Complex of Universidade Federal do Paraná (CHC-UFPR). The secondary objectives were to identify the association between postural deviations and body mass index (BMI), and to evaluate the relation between orthopedic alterations, age and sex.

## METHOD

This is a cross-sectional study, conducted at the UEP of CHC-UFPR from May 2018 to December 2018. In total, 101 patients aged between 7 and 17 years were selected, and agreed to participate in the study upon clarification and signing of the informed consent form by parents and of the informed assent form by patients over 12 years of age. Individuals with primary orthopedic diseases, genetic syndromes and history of treatment with medication that affect growth were excluded. Patients who accepted to be part of the project were evaluated on the day of their previously scheduled consultation with the pediatric endocrinologist. They were all interviewed and evaluated by the same examiner, who was trained by a physical therapist experienced in the methodology applied.

Weight was obtained while patients were barefoot and wearing light clothes, using a Filizola^®^ anthropometric scale with capacity of 150 kg and a scale of 100 g. The unit used for recording was kilograms (kg). Height was measured while the individual was standing, in the Frankfurt position, barefoot and without headdresses, using a wall-mounted stadiometer (Stadiometer Mode S100, Ayrton Corporation^®^, Prior Lake, Minnesota), with a 0.1-cm accuracy. After collecting these data, BMI was calculated using the WHO AnthroPlus software. During clinical evaluation, the patient was positioned in front of a wall simetrograph, formed by a 5 cm checkered panel, at a distance of 3 m from the examiner, and evaluated in the frontal, right lateral, left lateral and dorsal planes.

The objective was to evaluate cervical spine abnormalities (presence of protrusion or not), deviation of the shoulders to the left or right, increase in the angle between triangles formed by the medial border of the arm and forearm with the lateral border of the trunk—angle of Thales, spinal deviations described as normal (absence of deviations) or altered (presence of scoliosis, kyphosis and/or lordosis), hip rotation (present or absent), knees (symmetrical, valgus and varus), ankle rotation (present or absent) and occurrence of flat feet. Another variable analyzed was the intermalleolar distance (IMD), obtained by measuring the distance between the right and left malleolus in centimeters, with the patient in an upright and relaxed position (when smaller than 10 cm for males and smaller than 12 cm for females, the values were considered normal).^6,7^

After inspection, patients performed two dynamic tests. Initially, the Adam’s forward bend test, which consists of asking the individual to touch the floor with their hands while flexing the body forward without bending the knees, as the observer is positioned behind the patient to assess spinal abnormalities.^8,9^ The second test, called the tip-toe test, is aimed to the integrity of the Achilles tendon and the mobility of the subtalar joint; the patient is asked to stand on tiptoe for a few seconds; the expected result is that the plantar arch is evident, and when it is not visualized, the feet are classified as fixed flat.^10^

The measures of central tendency and dispersion were expressed as means and standard deviations (M±SD) for continuous variables of symmetrical distribution, and as medians, minimum and maximum values for those with asymmetric distribution. To estimate the difference between continuous variables with symmetric distribution, the Student’s t test was applied, while for those with asymmetric distribution, the Mann-Whitney tests and the Kruskal-Wallis analysis of variance (ANOVA) were used, with a post-hoc Mann-Whitney’s test. To estimate the difference between categorical variables, the Pearson’s chi-square test was applied. The Pearson’s correlation coefficient was calculated to estimate the association between categorical variables. Univariate logistic regression was applied to estimate the probability of a binary categorical event according to the distribution of a continuous variable. For all tests, a minimum level of significance of 5% was adopted, and the sample provided a test power of 95%.

## RESULTS

The study sample was formed by 101 individuals, 47 (46.5%) males and 54 (53.5%) females, with mean age of 11.5±2.3 months. Thus, 53.5% of them were classified as obese, 28.7% as eutrophic, and 17.8% as overweight. We found no statistical difference when comparing BMI according to sex (p=0.27) or Z score according to BMI (p=0.45—Mann-Whitney test). The distribution of classification according to BMI for males and females showed no difference (p=0.21—Pearson’s chi-square test).

Table 1 shows the distribution of frequency of alterations in body segments, significantly more frequent among patients classified as obese. When comparing obese and eutrophic patients, changes in the cervical spine (p<0.01), spine (p<0.001), hips (p<0.01) and shoulders (p<0.001) were seen in more than half of obese patients (62.5, 62.2, 79.9 and 55.4%, respectively). Knee alterations were more frequent among obese individuals (p<0.001) compared with eutrophic individuals. Values were obtained using Pearson’s chi-square test. There was no variation with age or sex (p>0.05).

**Table 1.** Distribution of changes in % by static clinical evaluation

	Eutrophic	Overweight	Obese
Head	–	16.6	27.7
Column	17.2	22.2	64.8
Hip	–	5.5	22.2
Shoulders	10.3	38.3	61.1
Right cut	–	22.2	24.0
Left cut	–	–	24.0
Right knee	3.4	11.1	51.8
Left knee	3.4	11.1	50.0
Right ankle	6.8	11.1	18.5
Left ankle	3.4	16.6	20.3
Right foot	17.2	27.7	57.3
Left foot	17.2	22.2	57.4

There was no difference in cervical spine, spine and hip changes according to age. However, the probability of occurrence of shoulder alterations was higher the higher the age in the three groups (p<0.001—logistic regression). Changes in the Thales angle were more common among overweight patients. Overweight individuals presented with changes in the Thales angle on the right (p<0.01), while the obese presented it on both sides (p<0.001), according to Pearson’s chi-square test. There was no difference according to sex (p>0.05). A higher probability of change in the Thales angle was found according to age, and the values obtained through logistic regression were: right (R), p=0.02; left (L), p=0.06.

Knee changes were more frequent among obese patients when compared to eutrophic patients (p<0.001—Pearson’s chi-square test). No variation was seen relating to age (R: p=0.22 and L: p=0.16) or sex (p>0.05—Pearson’s chi-square test). The frequency of alterations in the ankles did not differ between groups (p=0.32 and p=0.11—Pearson’s chi-square test). No variation related to age (R: p=0.12 and L: p=0.19) or sex (p>0.05) were seen.

Change in the feet was more prevalent among the obese when compared to the eutrophic patients (p<0.001—Pearson’s chi-square test). There was no difference according to sex (p>0.05). Probability of feet alterations was not higher according to age, although the significance level was borderline (right foot: p=0.11; and left foot: p=0.07—logistic regression).

In the three groups classified according to BMI, few patients presented alterations in the integrity of the Achilles tendon (p=0.64 and p=0.41—Pearson’s chi-square test). No variation was observed when considering age (R: p=0.73 and L: p=0.34—logistic regression) or sex (p>0.05).

**Graph 1.**
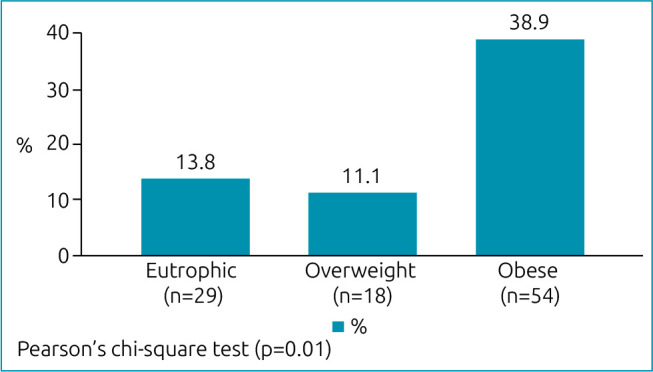
Frequency of change in Adam’s forward bend test according to body mass index.

As shown in Graph 1, a higher frequency of alterations was found during the Adam’s forward bend test among obese patients (p=0.01—Pearson’s chi-square test). There was no variation when considering age (p=0.23). The frequency was higher among males, with a borderline significance level (54.2% male vs. 26.7% female; p=0.07—Pearson’s chi-square test). Among patients with spinal alterations, a higher frequency of positive Adam’s forward bend test was observed in all three groups (p < 0.001).

There was an association between the MID measurement and changes in the right and left knees. The median MID among patients without knee alteration was 6.7 vs. 14.0 cm among those presenting with alteration (p<0.001—logistic regression). Measurements greater than 16 cm were associated with a probability of change of almost 100% (p<0.001—logistic regression).

The higher the BMI Z-score, the greater the MID measure. The frequency of MID alteration was higher among obese patients (p<0.01—Pearson’s chi-square test). There was no variation when considering age (p=0.13—logistic regression). The frequency of alterations was higher among males (70.8 vs. 27.7%; p<0.001—Pearson’s chi-square test).

## DISCUSSION

The literature describes a clear association between weight gain and the prevalence of comorbidities.^1^ When orthopedic alterations are analyzed, an association between increased BMI and a higher prevalence of axial skeleton deviations is seen in the studied population.

Spinal abnormalities are common in the pediatric population.^11^ The prevalence increases with increasing body mass. This association has been reported by several authors, including Kussuki et al. and Sibella et al.^12,13^ Sibella et all emphasizes that spinal abnormalities may result from a change in the center of gravity produced by the increase in abdominal volume and consequent adaptations of the spine. Relations between BMI and spinal abnormalities were also established, being represented by the variables: shoulder asymmetry, spinal inspection, Thales triangle and Adam’s forward bend test. Spinal abnormalities tend to decrease until adolescence, but the adolescents in this study had a higher percentage of joint abnormalities than younger patients.^14^

Displacement of the gravity center can lead to other skeletal adaptations, such as hip rotation, which can also be related to the data found in the patients in this study.^15^ It is noteworthy that the eutrophic group did not present hip abnormalities, corroborating the hypothesis that a protruding abdomen leads to adaptations of the pelvis. Similar data on the association between excess weight and anterior hip rotation were also found by Kendall et al. and Sato et al., who consider pelvic rotation in the pediatric population as an adaptive change in growth resulting from weakness of the paravertebral muscles and the rectus abdominis muscle.^10,16^ This tends to resolve itself around 12 years of age; in this study, there was no association between age and pelvis changes. We can infer that this association was not significant because the theory is based on eutrophic children, who do not present with excessive load on the musculoskeletal system, and it does not apply to the patients analyzed.

The changes described above trigger adaptations of the lower limbs to withstand the excessive load. As a consequence, knee valgus appears, which was the most common variation, as reported by Cicca et al.^17^ Results obtained both in the evaluation of the patient and in MID measurement in this study and in the work of Ciaccia et al. showed that adaptations are more frequent the higher the BMI.^18^ Ciaccia et al. evaluated children between 5 and 13 years of age and reported an equal distribution of patients with valgus knees between ages, similarly to the findings of our study.^18^

Comparison between studies that used MID as a parameter to determine the presence of knee valgus is difficult, as there is no standard established. The results can be interpreted based on different cut-off values and on the use or not of different measures between the sexes. In this study, we used the model adopted by Forlin et al. due to the similarity between populations.^6^

Other studies have shown, similarly to this investigation, that the obese pediatric population present flat feet more often than the eutrophic population.^19,20^ Mickle et al. suggest that this change stems from excess adiposity in the medial region of the foot, which would make it difficult to see the plantar arch.^20^ Other authors have reported changes in foot anatomy due to additional overload.^21^ In addition, the plantar arch undergoes physiological changes according to the age group, in a pattern similar to that of an adult when the child is between seven and ten years old, which means that flat feet can be physiological in a certain age group.^22,23^ This makes it difficult to establish whether patients with flat feet in clinical evaluation have this change due to high BMI or just as a result of normality variation. In this study, the prevalence of flat feet seems to increase with age, but the level of significance was borderline and did not allow to establish an association—a finding that differs from Pfeiffer et al’s.^24^

For the differential diagnosis of plantar arch diseases, the tiptoe test was used. A minority of patients had fixed flat feet, considered pathological. Tenenbaum et al.^19^ evaluated adolescents aged between 16 and 19 years and compared BMI with the clinical analysis of foot posture. They reported that for each increase of one point in BMI, the risk of having flexible flat feet increased by 5.4% in boys and by 4.7% in girls. On the other hand, the management of a patient with mobile flat feet is controversial, since a large portion of the population presents this alteration and no complications.^25^

This study had some limitations such as the lack of data on associated comorbidities, race, inheritance patterns of orthopedic diseases, pubertal stage, follow-up time in the service and assessment of physical activity level, data that could improve the quality of results obtained.

Obesity is associated with a greater number of osteoarticular changes in children and adolescents. The physician must introduce, in clinical practice, the osteoarticular assessment of overweight patients, in addition to a multidisciplinary follow-up, so that children and their families are instructed in the fight against obesity before the changes become dysfunctions and compromise Patients’ quality of life irreversibly.

## Approval by Institutional Ethics Committee

Universidade Federal do Paraná, CAAE: 76468017.0.0000.0096, opinion number: 2.629.135, from 27/03/2019
